# Genome packaging in multi-segmented dsRNA viruses: distinct mechanisms with similar outcomes

**DOI:** 10.1016/j.coviro.2018.08.001

**Published:** 2018-12

**Authors:** Alexander Borodavka, Ulrich Desselberger, John T Patton

**Affiliations:** 1 Astbury Centre for Structural Molecular Biology, School of Molecular and Cellular Biology, University of Leeds, Leeds LS2 9JT, UK; 2 Department of Medicine, Addenbrooke’s Hospital, University of Cambridge, Cambridge CB2 0QQ, UK; 3 Department of Biology, Indiana University, Bloomington, IN 47405, USA

## Abstract

Segmented double-stranded (ds)RNA viruses share remarkable similarities in their replication strategy and capsid structure. During virus replication, positive-sense single-stranded (+)RNAs are packaged into procapsids, where they serve as templates for dsRNA synthesis, forming progeny particles containing a complete equimolar set of genome segments. How the +RNAs are recognized and stoichiometrically packaged remains uncertain. Whereas bacteriophages of the *Cystoviridae* family rely on specific RNA–protein interactions to select appropriate +RNAs for packaging, viruses of the *Reoviridae* instead rely on specific inter-molecular interactions between +RNAs that guide multi-segmented genome assembly. While these families use distinct mechanisms to direct +RNA packaging, both yield progeny particles with a complete set of genomic dsRNAs.

## Highlights

• Multi-segmented dsRNA viruses package +RNAs prior to dsRNA synthesis.
• The three *Cystoviridae* +RNAs are sequentially packaged into a procapsid.
• *Reoviridae* assorted +RNA complexes are packaged during core formation.
• Sequence-specific RNA–RNA interactions mediate *Reoviridae* assortment.
• *Reoviridae* proteins that form viral factories facilitate RNA–RNA interactions.

**Current Opinion in Virology** 2018, **33**:106–112

This review comes from a themed issue on **Multicomponent viral systems**

Edited by **Stéphane Blanc** and **Yannis Michalakis**

For a complete overview see the Issue and the Editorial

Available online 23rd August 2018

**https://doi.org/10.1016/j.coviro.2018.08.001**

1879-6257/© 2018 The Authors. Published by Elsevier B.V. This is an open access article under the CC BY license (http://creativecommons.org/licenses/by/4.0/).

## Introduction

For viruses with segmented double-stranded (ds)RNA genomes, genome encapsidation requires recognition and packaging of a set of positive-sense (+) single-stranded (ss)RNAs in an environment rich in heterologous RNAs. Packaging may proceed by the sequential translocation of multiple +RNAs into pre-assembled procapsids [1,2^••^,^3^,4^••^], or, alternatively, by the cooperative assembly of a capsid shell around assorted RNA–RNA and RNA–protein complexes [5,6^•^]. For segmented dsRNA bacteriophages of the *Cystoviridae* family, packaging occurs via the helicase-driven insertion of three distinct +RNAs into preformed procapsids. By contrast, for viruses of the *Reoviridae* family, packaging likely begins with assortment of viral + RNAs to form an ordered complex that serves as the centerpiece for assembly of a surrounding capsid shell. Formation of the centerpiece appears to be driven by specific RNA–RNA interactions in a process chaperoned by viral non-structural RNA-binding proteins. Regardless of the packaging mechanism, viral + RNAs are only converted to dsRNAs by RNA polymerases located within procapsids, assuring coordination between RNA replication and capsid assembly.

## *Cystoviridae* RNA recognition and packaging

**Figure 1:**
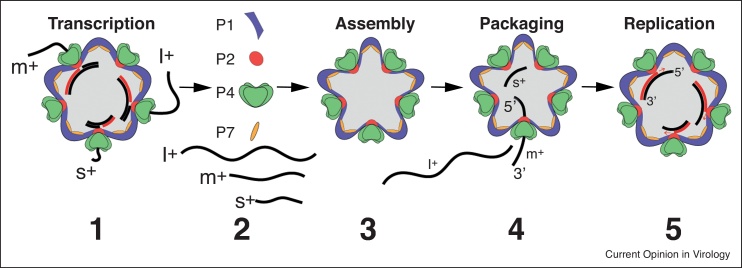
Assembly and pre-genome packaging in members of the *Cystoviridae* family. (1) Newly transcribed l+, m+, and s+RNAs are extruded from the procapsid. (2) Viral proteins P1 (core protein, purple), P2 (RdRP, red), P4 (RNA helicase/packaging motor, green), and P7 (assembly co-factor, yellow) co-assemble (3) forming new empty procapsids. (4) Assembled procapsids sequentially recruit RNAs, which are translocated inside the core by a hexameric packaging motor P4 in the order s+, m+, and l+. RNA packaging results in expansion of the procapsid. (5) After packaging, multiple + RNAs are replicated inside the procapsid by P2, forming dsRNAs.

*Source*: Adapted from Ref. [2^••^].

Members of the *Cystoviridae* share structural similarities with those of the Reoviridae [3]. Viruses of both families possess a *T* = 1 inner core that contains multiple copies of a viral RNA-dependent RNA polymerase (RdRP) [3,7]. Viral + RNAs packaged into these cores serve as templates for synthesis of dsRNA genome segments. The dsRNAs remain encased within the cores and, later in the viral lifecycle, are transcribed by core RdRPs to produce +RNAs [8, 9, 10]. The *Cystoviridae* are the only dsRNA viruses known to package +RNAs by translocation into pre-assembled cores [11]. The best studied member of the *Cystoviridae* is *Pseudomonas virus* φ6, whose assembly mechanisms have been examined using *in vitro* and *in vivo* systems [2^••^,4^••^,^8^,12^••^,13^••^]. The φ6 genome consists of three dsRNA segments: Large (L), Medium (M) and Small (S). Key to φ6 assembly is the formation of an empty, dodecahedron structure, termed a procapsid (Figure 1) [3,7]. The procapsid scaffold is a dodecahedral cage formed by 60 dimers of the core protein P1. Mounted on vertices of the procapsid is the hexameric RNA helicase P4 and bound to the interior face are the viral RdRP P2 and assembly factor P7 [2^••^,^14^]. The number of protein components in the φ6 procapsid vary, with particles containing 8–12 copies of P2, 5–6 copies of P4, and 30–60 copies of P7, suggesting a non-directed incorporation during assembly [15, 16, 17]. The role of the φ6 RdRP in packaging is limited, as viral + RNAs are translocated into procapsids that lack P2 [18]. Likewise, procapsids formed with the P4 S250Q mutant contain only ∼10% of the normal amount of the P4 helicase, yet package as efficiently as wild type procapsids, indicating that only a few copies of the helicase are necessary for +RNA translocation [19]. Remarkably though, procapsids with a single P4 helicase display altered packaging specificity, preferentially incorporating +RNAs of segments L and M, but not S [20].

## φ6 packaging signals

φ6 +RNAs (referred to as s+, m+, and l+ for segments S, M and L, respectively) are packaged sequentially into procapsids in order of size, beginning with the smallest. Critical to the process are segment-specific packaging (*pac*) signals present at the 5′ ends of φ6 +RNAs. Each *pac* signal resolves into two regions: a 5′ stretch of 18 nucleotides conserved among all three +RNAs and an adjoining downstream stretch of ∼200 nucleotides that folds to form a unique secondary structure [21,22]. φ6 packaging initiates with specific binding of the *pac* signal of the s+ RNA to a recognition site located on the vertices of the procapsid [18,23]. The P4 helicase destabilizes secondary structures on the s+ RNA, facilitating its 5′ > 3′ translocation into the procapsid [11,12^••^]. Insertion of the s+ RNA causes partial procapsid expansion, triggering conformational changes that generate a recognition site on the procapsid for the m+ *pac* signal [12^••^]. As a result, the m+ RNA is bound, and combined with the action of the P4 helicase, translocated into the particle. Packaging of the s+ and m+ RNAs results in additional procapsid expansion and conformational changes that expose a binding site for the l+ *pac* signal, leading to insertion of the l+ RNA (Figure 1). As a result of the sequential packaging of the s+, m+, and l+ RNAs, the procapsid becomes fully expanded, triggering the activation of procapsid RdRPs and the synthesis of S, M, and L dsRNAs [2^••^,^24^]. In summary, the specificity of φ6 RNA packaging is dependent not just on the presence of the P1 protein, but the conformational status of the P1 protein in context of the dodecahedral procapsid [3,4^••^,^7^].

Existing data indicate that φ6 utilizes a `headful' mechanism of RNA packaging, as both the type and amount of +RNA inserted into the procapsid are critical for display of *pac* recognition sites. For example, experiments performed using truncated s+ RNAs have shown that multiple copies of the RNA must be packaged into the procapsid to induce conformation changes sufficient for display of m+ *pac* site [12^••^,^25^]. Similarly, φ6 phage have been formed *in vivo* that contain two copies each of either M or L dsRNAs when provided with mutant m+ or l+ RNAs of half their normal size. Thus, these phage have genomes consisting of four dsRNA segments [25]. Remarkably, φ6 phage with a non-segmented genome can be formed by concatenating s+, m+, and l+ RNAs and including a 5′-terminal s+ *pac* signal [13^••^].

Despite this rather comprehensive understanding of the *Cystoviridae* packaging mechanism, many questions remain, including the nature of *pac* recognition sites on the procapsid and how their structure and activities are affected by RNA translocation. Auxiliary packaging mechanisms may exist, given the observation that some +RNAs lacking *pac* signals remain able to be packaged by φ6 and its close relative, φ8 [26]. Substitution of the *pac* signal on +RNA with a heterologous *pac* signal of another segment can result in the restoration of homologous *pac* sites in both segments through RNA recombination [26], indicating that additional mechanisms of selective RNA recruitment play a role in the packaging of these viruses.

## Reoviridae RNA recognition and packaging

Unlike *Cystoviridae*, members of *Reoviridae* carry out RNA packaging and replication in cytoplasmic inclusion bodies, termed viroplasms or viral factories [27, 28, 29, 30]. The components of viroplasms include structural and non-structural proteins essential for incorporation of complete sets of +RNAs into progeny cores. Although the φ6 packaging model reveals a mechanism by which segmented dsRNA viruses can acquire their genome, it is not compatible with the current knowledge of how *Reoviridae* progeny derive their 9–12 dsRNA genome segments. In order for the φ6 model to apply to the *Reoviridae,* up to 12 distinct conformational changes would have to occur to a preformed core structure during sequential packaging of +RNAs [12^••^]. However, no significant structural differences have been observed between *Reoviridae* empty and RNA-filled cores [31,32]. Indeed, studies with bluetongue virus (BTV, ten genome segments), mammalian orthoreovirus (MRV, ten), cytoplasmic polyhedrosis virus (CPV, ten), and rotavirus (eleven) suggest that, among the *Reoviridae*, viral + RNAs undergo assortment to form ordered complexes that nucleate the formation of a surrounding core protein (CP) layer. The cores of BTV and rotavirus are nearly indistinguishable, with copies of the viral RdRP and RNA capping enzyme (CAP) anchored to the interior core face near 5-fold vertices. By contrast, while MRV and CPV cores have similarly positioned RdRP molecules, their CAP components form turrets that extend through the core protein layer at 5-fold vertices.

## BTV and MRV RNA packaging

The only cell-free reconstitution system available to study the *Reoviridae* packaging mechanism was established for BTV [33^••^]. Results obtained using the reconstitution system [34] and a companion reverse genetics system indicate that BTV + RNAs assemble into larger RNA-rich complexes, possibly resulting from RNA assortment, *prior* to incorporation into core structures. In these experiments, efficient core assembly required all 10 BTV + RNAs and the presence of both, viral RdRP and CAP [33^••^]. Regions of BTV + RNAs important for segment-specific RNA packaging have been defined by reverse genetics. Such analysis revealed that packaging of BTV segment 9 +RNA requires up to 276 5′-terminal nucleotides and 393 3′-terminal nucleotides, suggesting that the 5′ and 3′ untranslated regions (UTRs) and parts of adjacent segment-specific coding regions contribute to the process [35]. Recent studies using oligonucleotides complementary to regions of BTV RNAs have also suggested that the 3′ UTRs and, to a lesser extent, the 5′ UTRs may be involved in inter-segment RNA–RNA interactions [36^•^].

Studies performed using MRV reverse genetics systems indicate that the 5′-terminal 125–200 nucleotides and 3′-terminal 180–285 nucleotides of MRV + RNAs are essential for RNA assortment and packaging [37^••^]. Because these sequences are substantially longer than the lengths of the UTRs of MRV + RNAs, packaging signals likely consist of both UTR and adjacent open reading frame (ORF) sequences, as was also observed with BTV + RNAs. Thus, unlike the findings for φ6 +RNAs, both ends of *Reoviridae* +RNAs are important for RNA packaging.

## Assembly of the rotavirus core

Studies on the rotavirus packaging mechanism have been challenging due to the lack of an *in vitro* packaging system and, until recently, the lack of a robust reverse genetics system. Nevertheless, substantial progress has been made in understanding various aspects of rotavirus RNA replication and particle assembly through structural and biochemical studies. Rotaviruses are triple-layered particles (TLPs), consisting of core, intermediate, and outer protein layers [9,38,39]. During cell entry, the outer capsid protein layer is lost, yielding double-layered particles (DLPs) that synthesize +RNAs [40,41]. Analysis of early replication intermediates isolated from rotavirus-infected cells [38] suggested that the viral + RNAs bind viral RdRP and probably CAP, forming complexes that are subsequently encased by CP [42]. Consistent with this model are the high-affinity, sequence-specific and the non-specific binding activities, respectively, of RdRP and CAP for rotavirus + RNAs. Assembly of a CP shell around RdRP/CAP/+RNAs complexes may be initiated by the binding of the RdRP to the inner surface of the core, through interactions with disordered N-terminal extensions protruding from the CP protein. Homotypic CP–CP contacts are likely to be enhanced and stabilized through additional high-affinity interactions of the CP N-terminal extensions with +RNAs, resulting in the cooperative assembly of the inner core [43^•^].

Single-molecule imaging of transcribing rotavirus DLPs has revealed that each genome segment is associated with a dedicated polymerase complex, responsible for synthesis and extrusion of a single type of +RNA from 5-fold channels of the particle [44^••^]. A similar arrangement of genome segments may exist for BTV, with each of its dsRNAs interacting with a specific polymerase complex positioned at a 5-fold vertex within the core [45]. Although details on the arrangement of dsRNA genome segments within the rotavirus and BTV particles are lacking, cryo-electron microscopy has provided considerable insight into the *in situ* organization of CPV genome segments. Its 10 segments are organized within the core in a non-symmetrical, non-spooled manner, with each interacting with a polymerase complex anchored on the inner surface near a 5-fold vertex. Two of the 12 vertices of the CPV core lack polymerase complexes, but are occupied with dsRNA emanating from genomic segments linked to polymerases bound to neighboring vertices [46^•^,47^•^].

**Figure 2:**
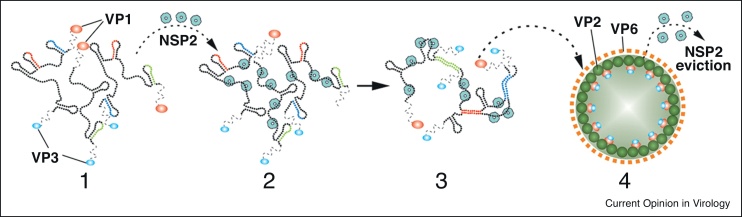
The proposed model of genome assortment and packaging in rotaviruses. (1) Within viroplasms, rotaviruses + RNAs bind viral RdRP (VP1, shown in red), and RNA capping enzyme (VP3, in blue), forming +RNA/VP1/VP3 complexes. (2) Binding of the octameric RNA-binding NSP2 (teal), causes structural remodeling of the viral + RNAs, exposing otherwise sequestered complementary sequences (sequences shown in red, blue and green). The complementary sequences promote base pairing between the different types of rotavirus + RNAs, a process representing RNA assortment. (3) The assorted RNA complex containing NSP2, VP1 and VP3, is predicted to nucleate VP2 core assembly (4). In this model, core assembly results in the displacement of +RNA-bound NSP2. RdRPs within new formed cores direct dsRNA synthesis, using +RNAs as templates (not shown).

*Source*: Adapted from Ref. [49^••^].

For rotaviruses, specific intermolecular interactions between distinct types of viral + RNAs may guide the assembly of an RNA assortment complex, prior to core encapsidation and RNA replication (Figure 2) [10,48,49^••^]. Unlike the φ6 RdRP, which alone can catalyse dsRNA synthesis from +RNA *in vitro* [14], the rotavirus RdRP lacks catalytic activity by itself. Instead, rotavirus RdRP is only active in the presence of CP, a phenomenon that allows rotavirus dsRNA synthesis to be tied to the packaging of +RNAs into newly formed cores [40,50]. Moreover, the mechanisms used to incorporate the rotavirus and φ6 RdRPs into cores are different. Notably, the rotavirus RdRP appears to be incorporated through its binding to +RNAs that are subsequently packaged in cores, while the φ6 RdRP is incorporated during procapsid assembly due to its affinity for the P1 core shell protein [15,16]. As a result, the number of rotavirus RdRP molecules in the core can be expected to equal the number of genome segments, while the number of the φ6 RdRP molecules can range from a few up to the number of vertices.

## Assortment of rotavirus +RNAs

The rotavirus nonstructural proteins NSP2 and NSP5 are non-specific RNA binding proteins that accumulate to high levels in viroplasms [28,51, 52, 53, 54, 55, 56]. NSP2 exists in infected cells as doughnut-shaped octamers that possess helix-destabilizing activity, while NSP5 is a serine-threonine rich phosphoprotein with a poorly understood multimeric status. NSP2 and NSP5 have affinities for core structural proteins, perhaps reflecting a role for NSP2 and NSP5 in recruiting core proteins to viroplasms or chaperoning the function of core proteins during RNA assortment, packaging and/or replication. Notably, NSP2 is a component of replication intermediates engaged in dsRNA synthesis [51].

Current models favor the idea that rotavirus assortment is driven by intermolecular interactions occurring between the 11 species of viral + RNAs [9,10,48,49^••^,57^••^]. For BTV, recent *in vitro* studies and reverse genetics experiments strongly support such a model [36^•^,^58^]. For rotaviruses, inter-segment RNA–RNA interactions have also been detected *in vitro.* Using two-color fluorescence correlation spectroscopy (FCCS)-based RNA–RNA interaction assays, NSP2 was found to promote *de novo* formation of sequence-specific inter-molecular contacts among the 11 rotavirus + RNAs [49^••^] (Figure 2). Inter-segment RNA hybridization was dependent on NSP2-mediated structural reorganization of +RNAs under conditions requiring a large molar excess of NSP2 [49^••^], which is likely achieved in viroplasms during infection. The study also suggested that although all 11 +RNAs could bind multiple copies of NSP2, only certain RNA subsets form stable, sequence-specific inter-molecular contacts. The redundancy of RNA–RNA interaction sites detected within the coding regions of individual +RNAs may explain how genetically diverse strains of rotaviruses maintain the potential to undergo reassortment [59], while retaining packaging selectivity due to the conservation of packaging signals located within the UTRs. The recently developed plasmid-only-based rotavirus reverse genetics system [60^••^] will be an important tool for testing predictions made about the assortment process using FCCS-based RNA–RNA interaction assays.

It remains unclear how rotavirus + RNAs are selected for packaging into cores. Studies with pre-formed core particles failed to provide evidence of preferential packaging of viral + RNAs *in vitro* [61], suggesting that other factors likely contribute to packaging specificity. One possibility is that the only +RNAs packaged into cores are those bound to the viral RdRP, a protein with high affinity for the 3′-terminus of rotavirus + RNAs. In this scenario, the inability of the RdRP to bind cellular mRNAs would preclude packaging of non-viral RNAs into cores. Moreover, the RdRP requirement would prevent the packaging of viral + RNAs that lack the necessary bound polymerase to drive RNA replication within the core [40]. Although signals that promote packaging into cores have not been identified in rotavirus + RNAs, it is interesting to note that rotavirus genome segments containing sequence duplications are preferentially packaged into progeny viruses relative to wild-type segments [62]. This observation suggests that duplicated sequences may contain an increased number of specific packaging signals, thus increasing their packaging efficiency.

It has also been suggested that global features of RNA genomes, for example, their relative compactness and overall size and shape, could be important for efficient encapsidation. By extrapolation, it may be that only assortment complexes containing the complete set of 11 rotavirus (+)RNAs may have the global features enabling packaging within a viral core [6^•^,^63^]. Binding of multiple copies of NSP2 to the 11 +RNAs could modulate core assembly, thus precluding uncontrolled core nucleation [64], and/or prepare the RNA assortment complex for encapsidation. It has been shown that NSP2 may play a role in preventing premature initiation of replication of the packaged +RNAs [65]. Although NSP2 can promote strand-annealing reactions between non-cognate complementary sequences [66], it appears that the RNA assortment in rotaviruses may be primarily attained via sequence-specific RNA–RNA interactions [49^••^,67^••^]. Importantly, non-cognate RNAs are outcompeted by cognate rotavirus RNA transcripts in RNA–RNA *in vitro* interaction assays in the presence of NSP2 [49^••^]. Accordingly, no rotaviruses containing host RNAs or lacking one of the gene segments have ever been reported to date. Exploring whether viroplasms can exclude non-cognate RNA, so that only RV + RNAs are retained inside for efficient interaction with each other, will provide clues about the high selectivity of genome packaging in these viruses. Numerous RNA-binding proteins have been shown to phase-separate in the presence of RNA to form liquid droplets, consisting of ribonucleoprotein granules containing multiple RNAs [68^•^,^69^,^70^]. Given the highly dynamic nature of viroplasms and their high protein and RNA content, it is possible that similar mechanisms may be involved in their formation and function in rotavirus-infected cells.

## Concluding remarks

The multi-segmented dsRNA viruses of the *Reoviridae* family employ mechanisms of RNA selection and packaging that are distinct from those utilized by members of the tri-segmented *Cystoviridae.* Such differences can be attributed to the different complexities of selecting and packaging 9–12 versus 3 +RNAs into progeny cores. In the case of *Reoviridae,* RNA selection and packaging take place in viroplasms, sites where +RNA and protein components are concentrated, including chaperone proteins that can act to facilitate and modulate such processes. The NSP2 octamer — an RNA-binding protein with a helix-destabilizing activity — is key for RNA selection and packaging in the case of the rotavirus. Notably, nonstructural proteins similar to NSP2 are produced by other members of the *Reoviridae*, suggesting a conserved essential role for NSP2-like proteins in the replication and packaging of all viruses in the family. Increasing evidence suggests that *Reoviridae* assortment is driven by interactions involving multiple regions of +RNAs, including those positioned within UTRs and even ORFs. Interestingly, RNA–RNA interactions that give rise to assortment are dependent on NSP2, and as a result appear likely to occur preferentially in viroplasms. Thus, free +RNAs in the cytosol may be precluded from interacting with each other, preventing the formation of RNA–RNA complexes that may interfere with translation or stimulate RNA-dependent innate immune responses. With the recent development of a completely plasmid-based rotavirus reverse genetics system it will become possible to identify sequences in +RNAs that direct the RNA–RNA interactions leading to complete assortment of all 11 viral + RNAs. From this should come information helping to explain how RNA assortment influences the maintenance of preferred genome constellations, a characteristic common for rotavirus strains isolated from different hosts and a factor influencing rotavirus evolution and genetic diversity.

## References and recommended reading

Papers of particular interest, published within the period of review, have been highlighted as

• of special interest
•• of outstanding interest
